# A longitudinal study of phenotypic changes in early domestication of house mice

**DOI:** 10.1098/rsos.172099

**Published:** 2018-03-07

**Authors:** Madeleine Geiger, Marcelo R. Sánchez-Villagra, Anna K. Lindholm

**Affiliations:** 1 Palaeontological Institute and Museum, University of Zurich, Karl-Schmid-Strasse 4, 8006 Zurich, Switzerland; 2 Department of Zoology, University of Cambridge, Downing Street, Cambridge CB2 3EJ, UK; 3 Department of Evolutionary Biology and Environmental Studies, University of Zurich, Winterthurerstrasse 190, 8057 Zurich, Switzerland

**Keywords:** commensalism, tameness, pigmentation, domestication syndrome, human, evolutionary rate

## Abstract

Similar phenotypic changes occur across many species as a result of domestication, e.g. in pigmentation and snout size. Experimental studies of domestication have concentrated on intense and directed selection regimes, while conditions that approximate the commensal and indirect interactions with humans have not been explored. We examine long-term data on a free-living population of wild house mice that have been indirectly selected for tameness by regular exposure to humans. In the course of a decade, this mouse population exhibited significantly increased occurrence of white patches of fur and decreased head length. These phenotypic changes fit to the predictions of the ‘domestication syndrome'.

## 1. Introduction

The outcomes of domestication in the diversity of breeds are familiar, but much less is known about the effects of the initial phases of the domestication process. These include alternative pathways: ‘commensal', ‘prey’ and ‘directed' [1–3]. The commensal pathway is characterized by wild animals, such as wolves, entering an anthropogenic habitat, and eventually becoming habituated and tame. The prey pathway concerns increasingly more intensively managed prey species. The directed pathway describes a process in which humans intentionally domesticate wild species, based on knowledge of previous domestication processes, such as mink and silver fox [4].

Although the distribution of traits across domesticated forms of mammalian species is not universal [5], some traits do commonly appear, irrespective of phylogenetic relatedness. The ‘domestication syndrome' includes the appearance of white patches of fur and a reduced relative brain and snout size. The leading mechanistic explanation is that selection for tameness results in developmental changes in the neural crest that produce this cascade of features [6]. Experimental selection for tameness in silver foxes [7], rats [8,9] and mink [10] have shown the power of the directed pathway of domestication. The commensal pathway of domestication remains unexplored. In this study, we describe phenotypic changes in a population of wild house mouse (*Mus musculus domesticus*) that has experienced an environment similar to that hypothesized for the early phases of the commensal pathway: frequent exposure to humans, without deliberate artificial selection. We investigate if domestication-associated traits that have been scored in this population (occurrence of white patches of fur and head length) are changing in this mouse population and compare the rate of evolution of these traits.

## 2. Material and methods

The study population was established in 2002 in an uninhabited barn in Illnau, Switzerland, with the introduction of 12 wild-caught individuals [11] trapped in cattle and sheep sheds at two neighbouring small working farms. Mice in these small working farms are considered as unwanted pests. The founder individuals reproduced successfully and the study population increased considerably in size and now comprises 250–430 individuals at any given time [12]. The floor area of the study barn is 72 m^2^, resulting in 3.5–6 mice m^−2^ in the barn, a density that is below the 10 mice m^−2^ that can occur in stable, commensal house mouse populations [13]. The sex ratio is approximately equal [11] and inbreeding levels did not increase over time in the study population [14]. Mice are free to enter and leave through numerous openings which are too small for predators to pass through (e.g. domestic cats, martens, foxes, owls). Other small rodents living outside occasionally enter but have never colonized the barn. The population is subject to diseases [15] and parasites. Commensal mice on farms typically have abundant food resources and a human-made environment [16], which is also the case in the study population. A standardized ad libitum feeding regime is followed with a 50 : 50 oat and commercial rodent food (Haefliger AG) mixture.

Owing to regular experimental handling of mice and monitoring of nests over 14 years, we hypothesize that these wild mice have been habituated and unintentionally selected for tameness for approximately 20 generations [14]. All nest-boxes and hiding places are monitored at least every 10–13 days. Every mouse is usually first handled during these controls and subsequently, a second time when pups reach 13 days of postnatal age (±1 day, see [11]; electronic supplementary material, table S1 for details) to record sex, body weight and head length. Some pups were first discovered at the age of 13 ± 1 days, and then were handled once rather than twice as a pup. Every seven weeks, on average, comprehensive capture events are conducted in which all pups are handled, and all subadults and adults are captured and inspected in jars [11]. During these events, newly mature adults are also handled, inspected for white patches of fur and implanted with transponders [11]. Thus, in an average mouse lifespan in the study population of 196 days (28 weeks) [14], mice are handled two to three times and captured an additional three to four times. Capture success of transpondered adults is about 80% per capture event, thus some mice will be captured less often. This contrasts with virtually no human handling at all in sympatric commensal house mouse populations. Handling procedures correspond to good laboratory practice, i.e. mice are held gently but firmly in the hand and no mice are injured during the handling procedure. Duration of handling varies with age and temper of individuals and comprises 1–2 min per individual.

**Figure 1.**
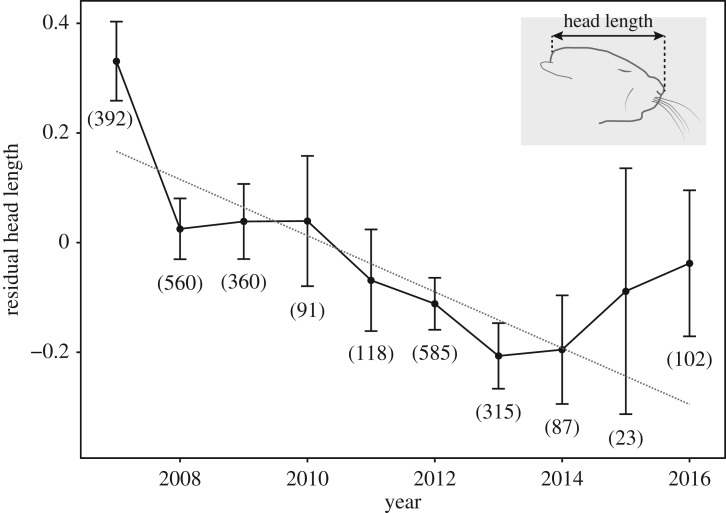
Decrease of relative head length of barn mouse pups. The dashed line shows the model predictions and error bars indicate 95% CI. The sample size per year is given in brackets. The box in the upper right corner shows the measurement of head length.

Two datasets inform the present study: (i) the occurrence of white patches and spots in the fur of adult mice from 2010 to 2016 (*n* = 2727) which was analysed with a binary logistic regression using white (1) and wild-type (0) as outcome and the date of monitoring (day/month/year) as the predictor variable. Approximately eight white hairs make up a small white spot. Individuals were systematically examined in the hand and tagged the first time they were caught as adults, here considered as 18 g or more [11], and therefore our dataset is restricted to first capture events of each adult (repeat examinations have been excluded). Only capture events with more than 10 recorded specimens were considered. (ii) Measurements of head length and body weight of 13-day-old (±1 day) mice from 2007 to 2016 (*n* = 2633), which were analysed with linear regressions. The age of pups was estimated by the developmental stage of hair growth, and ear and eye development. The eyes open at age 14 days [11]. Variation in age of measurement results mainly from non-daily visits to the field site. Head length of pups was measured in mm with digital callipers from the back of the skull to the tip of the snout (figure 1). Body weight (to the nearest 0.1 g measured by Mettler digital balances) was used as a proxy for body size. Pups of the same age found in the same nest were assigned a unique litter ID. To reduce variation in measurement error due to differences between observers, we only used measurements conducted by either of two highly trained people that collected data throughout the study period. Specimens described as runts were excluded. Time constraints in whole population capture events (in which up to 700 animals were handled in a day) precluded the measurement of head length in adults. Head length relative to body size (using residuals from least-squares regression of body weight on head length) was set as the response variable in a generalized linear mixed model with year of birth and sex as fixed effects and age, observer ID, litter ID and temperature as random effects. Age was used as a random effect because the age range of 2 days in the investigated mice (12–14 days old) could constitute a potential bias due to ontogenetic variation. Observer ID was included as a random effect because of possible systematic differences in measurements between observers. Litter ID was used as a random effect because measurements from individuals of the same litter are not independent due to a similar genetic, environmental and maternal background. Temperature (as measured at the day of pup examination in the barn) was used as a random effect because directed changes of temperature over the years might have had an influence on body weight and skull length [17–19]. The time intervals in these datasets differ because the initial purpose of the study was to investigate behaviour, disease transmission and genetics in house mice [15,20–24]: white spotting was very rare and, therefore, not systematically recorded before 2010 and head length information was not collected prior to 2007.

Evolutionary rates of head length change were calculated in darwins (*d*) [25] and haldanes (*h*) [26] (for a review, see [27]). Darwins were calculated as *d* = [ln(*x*_2_) − ln(*x*_1_)]/[*t*_2 _− *t*_1_], where ln(*x*_2_) − ln(*x*_1_) is the difference between the ln-transformed sample means of head length at time *t*_2_ and *t*_1_, and *t*_2_ − *t*_1_ is the elapsed time (in Myr) between *t*_2_ and *t*_1_. Head length changes (*x*_1_, *x*_2_) between 2007 (*t*_1_) and 2016 (*t*_2_) were calculated. Haldanes were calculated as $h=[\left(\text{ln}(x_{2})/s_{\text{ln}\;x_{2}})-(\text{ln}(x_{1})/s_{\text{ln}\;x_{2}}\right)]/g$, where ln(*x*_1_) and ln(*x*_2_) are the ln-transformed sample means of head length at the beginning and the end of the study period, respectively, $s_{\text{ln}\;x_{1}}$ and $s_{\text{ln}\;x_{2}}$ are the pooled standard deviations of ln(*x*_1_) and ln(*x*_2_), respectively, and *g* is the number of generations between the beginning and the end of the study period (years divided by generation length). Generation time in the study population has been estimated to be 263 days [14]. Species mean evolutionary rates of head and skull measurements in eight rodent species (and subspecies in the case of *Peromyscus maniculatus*) were used for comparison [28–31] (table 1). A one-sample Wilcoxon's signed-rank test was used to compare the median evolutionary rates in darwins from the literature to that of the study population. Statistical comparisons of haldane estimates could not be conducted because the literature data were only available for two species (table 1). Analyses were conducted using Microsoft Excel 2010, R v. 3.1.3 [32], and RStudio v. 0.98.1103 [33].

**Table 1.** Evolutionary rates of skull dimensions in different rodent populations. Only studies (references in brackets) on contemporary microevolution (15–60 years) using an allochronic study design (same population at different points in time [27]) were considered. n.a., not applicable.

species	study area	timeframe (years)	traits showing significant change	darwins (*d*)	haldanes (*h*)
*Akodon cursor* [28]	EPTEA Mato do Paraíso (Brazil)	15.5	least interorbital width	1622.03	n.a.
			height of skull	1127.18	n.a.
*Cerradomys subflavus* [28]	EPTEA Mato do Paraíso (Brazil)	15.5	rostrum width	2022.72	n.a.
			length of incisive foramina	4308.62	n.a.
*Oligorysomys nigripes* [28]	EPTEA Mato do Paraíso (Brazil)	15.5	nasal length	1945.81	n.a.
			rostrum length	1915.23	n.a.
*Peromyscus maniculatus* *anacapae* [29]	Anacapa (Channel Island)	38	intermeatus width	1730.00	n.a.
			breadth of rostrum	461.00	n.a.
			depth of braincase	702.00	n.a.
			length of incisive foramen	688.00	n.a.
			snout width	603.00	n.a.
			breadth of zygomatic plate	685.00	n.a.
*P. m. santacruzae* [29]	Santa Cruz (Channel Island)	38	intermeatus width	2682.00	n.a.
			depth of braincase	792.00	n.a.
*P. m. elusus* [29]	Santa Barbara (Channel Island)	44	length of nasals	916.00	n.a.
			depth of braincase	619.00	n.a.
*P. leucopus* [30]	Chicago (Illinois)	27.5	breadth of rostrum	2134	0.106
			depth of braincase	915	0.038
			greatest length of skull	1005	0.017
			length of braincase	1041	0.023
			length of incisive foramen	4772	0.125
			length of palate plus incisor	1631	0.035
			length from supraorbitals to nasals	1422	0.032
			zygomatic breadth	1527	0.041
*Rattus rattus* [31]	Anacapa (Channel Island)	60	zygomatic breadth	1912	0.145
			greatest length of skull	1892	0.060
			interorbital breadth	1183	0.433
			breadth of braincase	1470	0.192
			length of palate plus incisor	2567	0.127
			length of braincase	1953	0.098
			length of incisive foramen	2074	0.323
			depth of braincase	1084	0.209
summary (of species means)			average rate	1670	0.125
			median rate	1752	0.125
			minimum rate	768	0.052
			maximum rate	3166	0.198

## 3. Results

**Figure 2.**
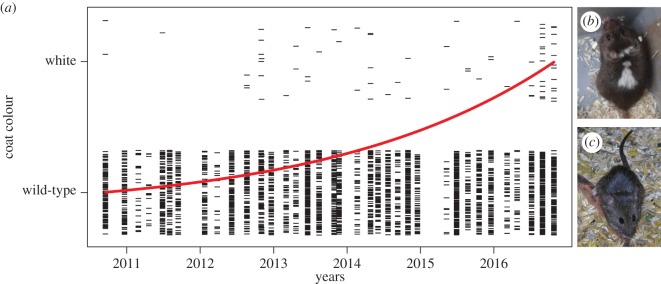
Increased occurrence of white spots and patches in the barn mice (*a*). ‘White' signifies specimens with white patches or spots (*b*) and ‘wild-type' signifies the usual brown coloration (*c*). The red line indicates a significant increase in the occurrence of white patches and spots from 2010 to 2016.

The proportion of adult mice with white patches of fur increased more than twofold from 2.5% in 2010 to 5.4% in 2016 (*z* = 4.61, *p* < 0.0001; figure 2). Head length in 13-day-old mice decreased significantly between 2007 and 2016 (*F*_1,2631_ = 195.00, *p* < 0.0001), as did body weight (*F*_1, 2631_ = 78.77, *p* < 0.0001). Head length relative to body weight also decreased significantly (*t* = −4.42, *p* < 0.0001, figure 1). Sex did not significantly influence head length (*t* = 1.59, *p* = 0.113). The random effects explained about 45% of the total variance (electronic supplementary material, table S2 and figure S1). Raw data are available as electronic supplementary material, S2 and S3.

Absolute head length decreased by 4017 darwins and 0.499 haldanes. Although the rate in darwins lies in the range of microevolutionary rates reported for some skull measurements in some rodent species (table 1), it is significantly higher (Wilcoxon signed-rank test, *V* = 0, *p* = 0.0078). The rate in haldanes exceeds the maximum values reported in the literature by more than twofold (table 1).

## 4. Discussion

The increase in the occurrence of white fur patches in adults (figure 2) and the decrease in absolute and relative head length and body size in young mice (figure 1) in our study demonstrates that within few generations detectable phenotypic changes can occur that are known from many different domesticated species and are part of the so-called ‘domestication syndrome' [6,7,34,35]. However, we cannot exclude that these phenotypic changes, consistent with selection for tameness, have arisen by other processes.

We hypothesize that our procedures of regular capture and handling of mice and exposure to humans have led to the dispersal of anthropophobic mice from the study population, while more anthropophilic ones have remained and bred (or possibly immigrated from other populations, which would suggest that the influence of the experimental set-up would be even stronger than inferred). If such tolerance towards human disturbance, an aspect of tameness, is heritable, then tolerance will increase over time. Tameness in domesticates is associated with a downregulation of the fear/stress system, specifically the hypothalamic–pituitary–adrenal axis and serotonin levels [6]. In laboratory mice, tameness is variable and influenced by genetic background [36,37]. A recent hypothesis [6] suggests that selection for tameness leads to reduced neural crest cell input which in turn gives rise to the ‘domestication syndrome', affecting stress and fight or flight responses as well as shortening the snout and causing white spots. The high evolutionary rates we report suggest that such selection can be strong. However, the link between tameness and white spotting is probably not straightforward given the lack of quantitative trait loci influencing both in rats [38].

Commensalism in *M. m. domesticus* is probably the result of human development of sedentary ways of life about 15 000 years ago in the Near East [39]. Subsequently, house mice spread towards Europe, following human migration, and reached western Europe (and Switzerland) less than 3000 years ago [40]. The house mouse populations of Switzerland are thus all descended from a lineage that can be considered anthropophilic (or even anthro-dependent [41]) and thus already habituated—to a certain degree—to interactions with humans, a first step on a commensal pathway towards domestication [1,2]. Commensal house mice have been described as an intermediate form between non-commensal wild and laboratory house mice [42]. They have been found to be less agonistic towards conspecifics than non-commensal house mice [43] and to have shorter skulls than non-commensal conspecifics [44,45]—as predicted by the ‘domestication syndrome’ hypothesis [5,6].

Alternative explanations for the observed phenotypic changes include altered environmental conditions [16,35], and inbreeding and genetic drift [34]. In this study population, interspecific competition and predation at nests and feeding sites can be excluded and population density is within the range of natural populations [12,13]. Thus, frequent interaction with humans and reduced predation are likely the most important factors that distinguish the study population from sympatric commensal house mouse populations [16]. The lack of predation may play a role in the occurrence of pigmentation-related domestication characters [46]: mutations leading to white patches of fur occur in the wild as well as under domestication but might be selected against in the wild and also in commensal populations, whereas the relaxed selection on these traits in domestication (and also in the barn) allows them to persist or increase. If reduced head length relative to body size is associated with decreased risk of predation, then predator release could also contribute to this phenotype change, but we know of no evidence for this scenario. Genetic drift and inbreeding are likely to influence genetic variation underlying white spots and reduced head length in house mice, including in the study population. However, the only analysis to date (2003–2008) showed no change in inbreeding level in the study population [14]. Genetic drift is a possible explanation for some or all of the changes in head length, especially for the later increases (figure 1). Furthermore, morphological alterations of the skull due to adaptations to different food items [47] in the study population (see Material and methods) relative to the source populations (probably spillovers from harvest and fodder on farms), cannot be ruled out as a source of the observed decrease in head length, but also cannot explain the reversed trend in later years. Further work is required to rule out these alternative explanations.

## 5. Conclusion

Previous studies on foxes [7] and rats [8,9] have shown that strong selection for tameness can be associated with the peculiar phenotypic changes that are typical for domesticated animals. Our study shows that unintentional selection for tameness is associated with the same phenotypic changes in a wild house mouse population within few generations.

## Supplementary Material

Supplementary Information 1

## Supplementary Material

Supplementary Information 2

## Supplementary Material

Supplementary Information 3

## Data accessibility

Data are available as electronic supplementary material, S2 and S3.
