# Correction: ‘A longitudinal study of phenotypic changes in early domestication of house mice’ (2018), by Geiger *et al.*

**DOI:** 10.1098/rsos.231829

**Published:** 2023-12-13

**Authors:** Madeleine Geiger, Marcelo R. Sanchez-Villagra, Anna K. Lindholm

*R. Soc. Open Sci.* **5**: 172099 (Published online 7 March 2018). (https://doi.org/10.1098/rsos.172099)

We would like to report an erroneous interpretation of the equation and therefore calculation of the evolutionary rate estimate of *haldanes* in our article.

In his original definition, Philip Gingerich described the estimate of evolutionary rates in *haldanes* as:

$${}^{\prime }\mathrm{rate}\;(h)=\frac{\left[\left(\ln x_{2}/s_{\ln x})\;\text{–}\;(1\ln x_{1}/s_{\ln x}\right)\right]}{t_{2}\;\text{–}\;t_{1}},$$

where In *x*_1_ and In *x*_2_ are representative natural log [In)] measurements or sample means of ln measurements at times *t*_1_ and *t*_2_, respectively, and *s*_ln*x*_ is the pooled standard deviation of the In *x*_1_*s* and In *x*_2_s, respectively’. (Gingerich [1], p. 457)

In our paper, we misinterpreted the final word ‘respectively’ as a hint to use the individual standard deviations of each sample In *x*_1_ and In *x*_2_, instead of the pooled standard deviation of both, In x_1_ and In *x*_2_. In fact, we calculated haldanes as *h* = [(ln *x*_2_/*s*_ln*x*2_) – (1ln *x*_1_/*s*_ln*x*1_)]/*t*_2_ − *t*_1_.

We were made aware of this mistake by the reviewer of another article and subsequently scrutinized and re-analysed the data of earlier articles, which applied this equation.

For the corrected calculation of *haldane* estimates, we calculated the pooled standard deviation as S_ln*x*_ = [(SS_ln*x*1_ + SS_ln*x*2_)/(*n*_1_ − 1 + *n*_2_ − 1)]^0.5, where SS_ln*x*1_ and SS_ln*x*2_ are the sums of squares and n_1_ and n_2_ are the sample sizes of In *x*_1_ and In *x*_2_, respectively. Subsequently, *haldane* estimates were calculated as described in the original equation as given by Haldane (1993, p. 457).

While our original calculation resulted in a decrease in head length by 0.499 standard deviations per generation (*haldanes*) in the investigated house mouse population, the corrected calculation resulted in an estimated decrease by 0.062 standard deviations per generation. While the published, erroneous rate is much higher than previously reported rates of evolution of skull dimensions in different rodent populations on a contemporary scale (15–60 years), the new, correct rate lies within the rate of these literature records (original article, table 1).

The estimate of evolutionary rate in *darwins* is, however, not affected by this mistake. The *darwin* estimate has been found to lie within the range of literature records (table 1), but is statistically significantly higher than their median (see original article). Thus, the relatively large *darwin* estimate contrasts with the now average *haldane* estimate. We are aware of the advantages and drawbacks that both estimation methods entail (see e.g. Hendry & Kinnison [2]) and therefore regard both estimates as complementary.

Thus, despite the change of our *haldane* estimate due to the changed calculation, our conclusion concerning the rates of evolution in human proximity in this particular case remains the same: selection acting on animals in close proximity to humans can be strong and evolutionary rates can therefore be fast.

Change of paper:

Absolute head length decreased by 4017 darwins and 0.062 haldanes. Although the rate in darwins lies in the range of microevolutionary rates reported for some skull measurements in some rodent species (table 1), it is significantly higher (Wilcoxon signed-rank test, *V* = 0, *p* = 0.0078). The rate in haldanes lies within the range of values reported in the literature (table 1).
