# Supplementary material for: A longitudinal study of phenotypic changes in early domestication of house mice
Source: R Soc Open Sci. 2018 Mar 7;5(3):172099. doi: 10.1098/rsos.172099 (PMC5882729; doi:10.1098/rsos.172099)
Supplement: Supplementary Information 1 [file rsos172099supp1.docx]

**Royal Society Open Science**

**Supplementary Information 1**

**A longitudinal study of phenotypic changes in early domestication of** **house mice**

Madeleine Geiger^*^, Marcelo R. Sánchez-Villagra, Anna K. Lindholm^*^

**Supplementary Table S1. Absolute individual age of examined specimens**. Specimens were between 12 and 14 days of age when head length was measured, most were 13 days old.

| **Age (days)** | **Number of specimens** |
| --- | --- |
| 12 | 635 |
| 12.5 | 55 |
| 13 | 1389 |
| 13.5 | 57 |
| 14 | 497 |

**Supplementary Table S2. Random effects of the mixed effect model of residual head length.** Age, the investigated pups were between 12 and 14 days old when head length was measured; litter ID, identifies the pups belonging to the same litter (found together in same location and are of the same age); observer ID, identifies the two researchers who conducted the measurements; temperature, as measured in the barn at the day of examination of the pups. ‘Residual’ denotes the variation that is not due to one of the investigated effects; sd, standard deviation.

| **Groups** | **Variance** | **sd** | **% variance explained** |
| --- | --- | --- | --- |
| Age | 0.07 | 0.26 | 19.62 |
| Litter ID | 0.08 | 0.29 | 8.53 |
| Observer ID | 0.01 | 0.08 | 15.79 |
| Temperature | 0.04 | 0.19 | 1.47 |
| Residual | 0.23 | 0.48 | 54.58 |
| **Total variance** | 0.43 | - | 100 |





**Supplementary Figure S1. Relationships between random effects and residual head length in the here investigated mouse pups.** Age, the investigated pups were between 12 and 14 days old when head length was measured; litter ID, identifies the pups belonging to the same litter (found together in same location and are of the same age); observer ID, identifies the two researchers who conducted the measurements; temperature, as measured in the barn at the day of examination of the pups.
